# Ergonomic interventions for work in a sitting position: an integrative review

**DOI:** 10.47626/1679-4435-2023-770

**Published:** 2023-04-18

**Authors:** Camila Soares, Suraya Gomes Novais Shimano, Patrícia Ribeiro Marcacine, Luciane Fernanda Rodrigues Martinho Fernandes, Laianne Liliane Pereira Troncha de Castro, Isabel Aparecida Porcatti de Walsh

**Affiliations:** 1 Programa de Pós-Graduação em Fisioterapia, Universidade Federal do Triângulo Mineiro (UFTM), Uberaba, MG, Brazil; 2 Departamento de Fisioterapia Aplicada, UFTM, Uberaba, MG, Brazil; 3 Programa de Pós-Graduação em Atenção à Saúde, UFTM, Uberaba, MG, Brazil

**Keywords:** occupational health, pain, sitting position, ergonomics, saúde do trabalhador, dor, postura sentada, ergonomia

## Abstract

The sitting position is one of the most common positions in the workplace and one that can contribute to overloading the musculoskeletal system. Ergonomics can play a significant role in ensuring an appropriate relationship between people and their work and in achieving better conditions for workers’ health. The objective of this study was to consult the available evidence on the results of different ergonomic interventions for the musculoskeletal systems of workers who perform their jobs in a sitting position. This was an integrative review, searching the LILACS, MEDLINE, PubMed, SciELO, and CINAHL electronic databases for articles published from 2010 to 2019. The following keywords were used: Trabalhadores OR Workers OR Trabajadores AND Dor OR Pain OR Dolor AND Postura Sentada OR Sitting Position OR Sedestación AND Ergonomia OR Ergonomics OR Ergonomía. A total of 183 articles were identified, 14 of which were selected for the review. For qualitative analysis, the articles were organized by author, year, sample/population, objective, analytical instrument, intervention and type of intervention: combinations of physical exercise programs and postural and ergonomic guidance; different types of guidance and facilitating instruments; or configuration of furniture and use of supporting devices. A quantitative analysis of study quality was conducted using the Physiotherapy Evidence Database, based on the Delphi list. The interventions contributed to improve physical conditions and the tasks being carried out, making them more appropriate for the workers.

## Abstract (pt)

A postura sentada é uma das mais adotadas nos ambientes de trabalho e pode contribuir na sobrecarga do sistema musculoesquelético. A ergonomia pode apresentar um papel significativo para manter a relação adequada do homem com o trabalho e para abordar melhores condições à saúde dos trabalhadores. O objetivo deste estudo foi verificar as evidências disponíveis sobre os resultados de diferentes intervenções ergonômicas no sistema musculoesquelético de trabalhadores que exercem atividades na postura sentada. Tratou-se de uma revisão integrativa, utilizando as bases de dados eletrônicas LILACS, MEDLINE, PubMed, SciELO e CINAHL, com busca de artigos publicados no período de 2010 a 2019. Utilizaram-se os seguintes descritores: Trabalhadores OR Workers OR Trabajadores AND Dor OR Pain OR Dolor AND Postura Sentada OR Sitting Position OR Sedestación AND Ergonomia OR Ergonomics OR Ergonomía. Foram encontrados 183 artigos, sendo 14 selecionados. A análise qualitativa organizou os artigos de acordo com autor, ano, amostra/população, objetivo, instrumento de análise, intervenção e tipo de intervenção: associação de programas de exercícios físicos e orientações posturais e ergonômicas; diferentes formas de orientação e instrumentos facilitadores; e configuração do mobiliário e utilização de dispositivos auxiliares. A análise quantitativa da qualidade dos estudos considerou a escala Physiotherapy Evidence Database, baseado na lista Delphi. As intervenções contribuíram para melhorar as condições físicas e as tarefas executadas, tornando-as mais apropriadas para os trabalhadores.

## INTRODUCTION

Musculoskeletal disorders that compromise occupational health can be caused by the type of work activity, by heavy and repetitive physical work, by unhealthy positions, by prolonged activities, and by irregular characteristics of the workplace.^1,2^

The sitting position is one of the most common positions in the workplace, is a factor that causes disharmony between the body’s mechanical components, and can contribute to overload of the musculoskeletal system.^3,4^ Remaining sitting continually can cause muscle fatigue, reduce body proprioception, overload the body’s structures, and injure workers.^5^ As such, a healthy workplace should enable workers to vary their position.^6^

To achieve this, it is important to recognize the workplace, identify its risk factors, use ergonomic resources to prevent occupational diseases, and provide treatment and rehabilitation for those already affected.^7,8^

The objective of ergonomics is to improve working conditions through evaluation of tasks, projects, products, the working environment, and workers’ skills and limitations, studying and assessing the workplace’s limitations and the adaptations that can be made to it.^2,9^ Ergonomic interventions are essential to ameliorate the effects of sitting for long periods while working, improving the environment, the way work is organized, the tasks performed while working, and, as a consequence, workers’ comfort, safety, and wellbeing, making a positive contribution to their health.^5,9^ As such, employers have a duty to seek ergonomic resources to improve working conditions.^10^

The objective of this study was to consult the available evidence on the results of different ergonomic interventions for the musculoskeletal systems of workers who perform their jobs in a sitting position.

## METHODS

An integrative review was conducted. This is a method for collecting, assessing, and synthesizing the results of research on a specific subject. The study comprised six steps: formulation of the research question; sampling or searching for studies in the literature; extraction of data; assessment of the selected studies; interpretation of the results; and presentation of the review.^11,12^

The PICO (P: population; I: intervention; C: control or comparison; and O: outcome) strategy was employed to support formulation of the question and definition of the research problem,^13^ where P is people who work sitting position, I is ergonomic interventions, C is the workplace, and O is publications in the literature dealing with the subject. The following research question was adopted: “What does the literature report about ergonomic interventions and which ones can yield improvements for the musculoskeletal system of people who work in a sitting position?”

Searches were run on the following electronic databases for scientific studies published in Portuguese, English, or Spanish from 2010 to 2019: Literatura Latino-Americana e do Caribe em Ciências da Saúde (LILACS), Medical Literature Analysis and Retrieval System Online (MEDLINE), PubMed (both North-American), Biblioteca Eletrônica Científica On-line (SciELO), and Cumulative Index to Nursing and Allied Health Literature (CINAHL).

Keywords in Portuguese, English and Spanish from the Descritores em Ciências da Saude (DeCS) were combined using Boolean operators (AND and OR), as follows: (“Trabalhadores” OR “Workers” OR “Trabajadores”) AND (“Dor” OR “Pain” OR “Dolor”) AND (“Postura Sentada” OR “Sitting Position” OR “Sedestación”) AND (“Ergonomia” OR “Ergonomics” OR “Ergonomía”).

Inclusion criteria were articles published from 2010 to 2019 that investigated ergonomic interventions and used randomized clinical trial or quasi-experimental methodology. Exclusion criteria were articles and other publications that did not cover the chosen subject, review articles, duplicate articles, books, theses, dissertations, and undergraduate final-year projects.

Studies were classified during search, selection, and analysis by a single appraiser who conducted the electronic database searches independently. The studies identified were recorded and duplicates and those that did not meet the inclusion criteria were excluded.

Articles were selected in stages. First the titles were read, then the abstracts were read, and if they met the review objectives the full texts were then read. Data were analyzed with both quantitative and qualitative approaches.

Quantitative analysis of the methodological quality of articles was conducted using the Physiotherapy Evidence Database (PEDro) scale, which is based on the Delphi list.^14^ Each article was analyzed for internal validity and statistical interpretation and scored on the scale up to a maximum of 10 points.

The Delphi list assesses the external validity and potential for generalization or applicability of clinical studies, but this criterion is not counted in the final score. High methodological quality is defined as scores greater than or equal to seven. To be awarded points, studies must describe the following: origin or the sample and inclusion criteria (item 1), random allocation of subjects (item 2), concealed allocation (item 3), paring of the sample (item 4), blinding of subjects, therapists, and evaluators (items 5, 6, and 7), key outcomes measured in 85% of the sample at different times (item 8), participants receiving treatment or a control condition (item 9), and intergroup comparisons (item 10). The results of intergroup statistical comparisons were described for at least one key outcome.^14^

Studies were scored by two evaluators independently. Cases of disagreement were reassessed in conjunction until the evaluators reached a final consensus score.

The qualitative analysis comprised the following steps: 1) organization of articles by author, year, sample/population, objective, analytical instrument, intervention, and outcome; and 2) organization of articles according to the type of ergonomic intervention. In this step, for didactic reasons, the studies were allocated to different categories, depending on the type of intervention and considering the different options available for ergonomic intervention.

## RESULTS

The database searches were conducted in January 2020. The searches identified 183 articles, two of which were excluded because they were duplicates, leaving 181 articles to be analyzed by title, abstract, and inclusion and exclusion criteria. Fourteen of these articles were selected because they met the inclusion criteria and dealt with the chosen subject.

Table 1 lists the scores for the methodological quality of the clinical trials and quasi-experimental studies analyzed with the PEDro scale.^14^ According to the cutoff criterion, four studies were of high methodological quality with final scores of 8^15^ or 9.^16^ ^18^All other studies scored less than 7.^19-28^

**Table 1:** Methodological study quality of quasi-experimental studies and clinical trials according to the Physiotherapy Evidence Database (PEDro) scale^14^

Studies	PEDro										
	1	2	3	4	5	6	7	8	9	10	Total
Martins et al.^19^	1	0	0	0	0	0	0	1	0	1	3
Bazazan et al.^16^	1	1	0	1	1	1	1	1	1	1	9
Ailneni et al.^20^	1	0	0	0	0	0	0	1	1	1	4
Yoo & Park^21^	1	0	0	0	0	0	0	1	1	1	4
Levanon et al.^17^	1	1	1	1	0	1	1	1	1	1	9
Robertson et al.^22^	1	1	1	0	0	0	0	1	1	1	6
Ognibene et al.^18^	1	1	1	1	0	1	1	1	1	1	9
Dutta et al.^23^	1	0	0	1	0	0	0	1	1	1	5
Thorp et al.^24^	1	1	0	1	0	0	0	1	1	0	5
Pillastrini et al.^15^	1	1	1	1	0	1	1	1	1	1	8
Smith et al.^25^	1	0	0	0	0	0	0	1	1	0	3
Joines et al.^26^	1	1	1	1	0	0	0	0	1	1	6
Hayes et al.^28^	1	0	0	0	0	0	0	1	1	1	4
Aghilinejad et al.^27^	1	0	0	0	0	1	1	1	1	0	5

1 = eligibility criteria were specified; 2 = subjects were randomly allocated to groups; 3 = allocation was concealed; 4 = the groups were similar at baseline regarding the most important prognostic indicators; 5 = there was blinding of all subjects; 6 = there was blinding of all therapists who administered the therapy; 7 = there was blinding of all assessors who measured at least one key outcome; 8 = measures of at least one key outcome were obtained from more than 85% of the subjects initially allocated to groups; 9 = all subjects for whom outcome measures were available received the treatment or control condition as allocated or, where this was not the case, data for at least one key outcome was analyzed by “intention to treat”; 10 = the results of between-group statistical comparisons are reported for at least one key outcome.

Four studies had high methodological quality, scoring 8 or 9 points^15-18^; two articles were rated as of regular quality, scoring 6 points^22,26^; and eight articles had lower scores: three scored 5 points,^23,24,27^ three scored 4 points^20,21,28^ and two scored 3 points.^19,25^

With regard to study characteristics 1 study was conducted in Brazil and published in a Brazilian journal^19^ and 3 were 13 international studies.^15-18,20-28^ One of the studies was an uncontrolled clinical trial^28^; 7 were quasi-experimental studies^19,20-22,24,25,27^; and 5 were randomized, controlled clinical trials.^15-18,23^

Three studies investigated samples of more than 100 workers.^15,16,27^ Both men and women were studied in the majority of interventions,^17,18,20,24-26,28^ 3 studies had all-male samples,^16,21,27^ and 4 studies did not report sex.^15,19,22,23^

With regard to duration of the interventions, 2 studies conducted interventions lasting 6 months^26,28^; 2 interventions lasted 3 months^18,23^; and the longest intervention was 36 months.^15^

Assessment instruments were used to analyze pain or discomfort^15-18,22,24-28^; fatigue^16,24,26^; incapacity^18,19,27^; risk factors related to position, load or force, repetitiveness and frequency of changes of position, and effort^15,16,20,22,26^; and subjective assessments of perceptions of work.^17,19,22-26^ There were also assessments based on ergonomic analyses,^19^ anthropometric measurements,^20^ markers to capture movements,^20^ surface electromyography,^17,21^ ultrasonic three-dimensional movement sensing,^21^ and lighting level measurement.^26^

The findings related to the ergonomic interventions are presented under the following subheadings: combinations of physical exercise programs and postural and ergonomic guidance^19^; different types of guidance and facilitating instruments^16,17,20,21^; configuration of furniture^15,18,22-24^; and use of auxiliary devices that could interfere with position and with execution of work tasks.^25-28^

Charts 1, 2, 3, and 4 list the main results of each of the studies, by author, year of publication, sample, study design, objective, analytical instruments, interventions, and outcomes.

**Chart 1:** Characteristics of studies in the category: combinations of physical exercise programs and postural and ergonomic guidance

Authors (year)	Sample (n)	Study design	Objective	Analytical instrument	Intervention	Outcome
Martins et al.^19^ (2011)	11 office workers working 8 hours per day, using computers	Longitudinal, prospective, quasi-experimental study,	To assess the effects of a program of physical exercise and postural and ergonomic guidance on musculoskeletal complaints and job satisfaction of office workers	Ergonomic data collection and analysis; the Couto Ergonomics Survey questionnaire: for perception of job role; neck dysfunction index; and Occupational Stress Indicator (OSI): to assess job satisfaction	The program of stretching exercises and guidance relating to physical posture and furniture used at the workstation was administered for 5 weeks	The number of complaints of discomfort/pain reported by the office workers was reduced after the intervention program. No association was observed between number of complaints and job satisfaction.

**Chart 2:** Characteristics of studies in the category: different types of guidance and instruments to facilitate implementing guidance

Authors (year)	Sample (n)	Study design	Objective	Analytical instrument	Intervention	Outcome
Bazazan et al.^16^ (2019)	188 male office workers (operatives from a petrochemical control room) allocated to an intervention group (91) or a control group (97)	Randomized controlled clinical trial	To examine the effect of a posture correction-based intervention on musculoskeletal symptoms and fatigue	NMQ: musculoskeletal symptoms; MFI-20: Multidimensional Fatigue Questionnaire; RULA: to assess posture	Use of biofeedback to transmit communication via an audible and vibration alert to help workers to improve their posture when working	The low-cost device proved of considerable benefit for improving posture and reducing musculoskeletal symptoms and fatigue.
Ailneni et al.^20^ (2019)	19 workers: 10 women and 9 men	Quasi-experimental study	To evaluate the effect of a wearable posture sensor on posture and on physical demands on the head and neck during office work	Anthropometric measurements, reflective markers on several parts of the body, the floor, the chair, the table edge, and the laptop; infrared cameras: movement capture	Participants performed typing tasks with and without the sensor, sitting and standing up and were allowed to adjust their workstations during the experiment using a psychophysical method	The wearable sensor reduced postural stress on the neck. The effect was more significant when using the standing workstation compared to the sitting workstation.
Yoo & Park^21^ (2013)	12 workers (men)	Quasi-experimental study	To investigate the difference in kinematics of the neck and trunk segments as well as muscular activation between conditions with and without posture related auditory cueing	Surface electromyography: to measure the activity of the erector spine and upper trapezius; ultrasonic three-dimensional movement; CMSMS: to capture kinematic data, record angles of front and trunk during work, in the sagittal plane	A posture related auditory cueing (PAC) program, that played a cue consisting of a beep followed by a spoken postural correction suggestion at 300 second intervals	The auditory cueing program was positive for preventing unhealthy postures in the workplace and was recommended for practical use in the workplace.
Levanon et al.^17^ (2012)	66 workers (23 men and 43 women) allocated to a group with biofeedback (22) or a group without biofeedback (23) and control group without intervention (21)	Randomized controlled clinical trial	To test the efficacy of a workplace intervention for reducing musculoskeletal complaints among computer workers.	NMQ: to assess pain symptoms; RULA: to assess risk factors of posture and forced effort of arms, trunk, and legs; DCSQ: for psychosocial assessment of psychological demands, attitudes, and support in the workplace; SEMG: to report muscle activity	Three intervention programs: ergonomic intervention, including biofeedback and surface electromyography, intervention without biofeedback, and a control group	The interventions were effective for reducing workers’ musculoskeletal complaints and pain. The intervention with biofeedback had no unique contribution in comparison to other groups.

CMSMS = ultrasonic movement analysis system; DCSQ = Swedish Demand Control and Support Questionnaire; SEMG = surface electromyography; MFI-20 = Multidimensional Fatigue Inventory; NMQ = Nordic Musculoskeletal Questionnaire; RULA = Rapid Upper Limb Assessment.

**Chart 3:** Characteristics of studies in the category: configuration of furniture

Authors (year)	Sample (n)	Study design	Objective	Analytical instrument	Intervention	Outcome
Robertson et al.^22^ (2017)	82 workers allocated to a control group (42), an intervention group (14) or an intervention plus training group (26)	Longitudinal, quasi-experimental, controlled study	To examine workers’ computing behaviors, postures, and musculoskeletal discomfort, and their relationship to psychosocial factors.	OEA: for assessment of the ergonomic configuration of the workplace and general items under workers’ control; RULA: to assess the physical load of working postures, muscle exertion, and musculoskeletal risks; ergonomic atmosphere scale: to understand how management respond to workers’ ergonomic needs; corporate communication culture: to assess workers’ sense of community and knowledge about their job roles; Corlett & Bishop scale: to assess musculoskeletal symptoms	Macroergonomic intervention with flexible workplace design (environmental changes), and office ergonomics training	The intervention was beneficial for workplace organization, for postures, for reducing musculoskeletal symptoms, and for improving workers’ psychosocial conditions.
Ognibene et al.^18^ (2016)	46 workers allocated to an intervention group (23) or a control group (23)	Randomized controlled clinical trial	To determine whether chronic low back pain in office employees might be attenuated through the introduction of a sit-stand workstation	RMDQ questionnaire: to assess extent of physical incapacity caused by low back pain; modified BPI: to measure level of pain and extent to which it interferes with aspects of life, modified by addition of questions about time spent sitting down at work and pain medication	Introduction of a sit-stand workstation with instructions on how to use it correctly and comfortably	Chronic low back pain was reduced by introduction of a sit-stand workstation in an office environment.
Dutta et al.^23^ (2015)	25 office workers allocated to a supervisors group (8), a no-supervisors group (10), or a control group (7)	Randomized controlled clinical trial	To gain knowledge about participants’ experience and perceptions of a workplace intervention involving the introduction of sit-stand desks.	Individual interviews: to assess benefits and disadvantages of using the desks, their impact on health and on interaction with coworkers; and focus groups for data collection on participants’ and non-participants’ experiences in relation to the ways that the workplace was changed to introduce the desks, with an extension for interactions with coworkers, perceptions of productivity, and physical health-related changes.	Intervention with desks that offer height adjustments to enable workers to work sitting or standing; weekly reminders by e-mail, anti-fatigue mats, and ergonomic assessment	There was a high level of satisfaction with the sit-stand desks. Some workers reported relief of low back pain, improved posture and energy, and greater interaction with coworkers.
Thorp et al.^24^ (2014)	23 workers (17 men and 6 women)	Quasi-experimental study	To examine whether using a height-adjustable workstation can improve subjective levels of fatigue, musculoskeletal discomfort and work productivity relative to seated work.	A modified version of the questionnaire CIS20-R strength questionnaire and the MAF scale: to assess fatigue; NMQ: to assess musculoskeletal discomfort; modified version of the HWQ: to assess work productivity; 10 cm VAS: to assess acceptability of the adjustable workstation	Use of an electric ergonomic workstation (sit-stand condition) or working in a sitting position (sit condition)	Transitioning from sitting to standing every 30 minutes led to a significant reduction in fatigue levels and back discomfort in overweight workers, while maintaining work productivity.
Pillastrini et al.^15^ (2010)	153 workers allocated to an intervention group (80) or a control group (73)	Randomized controlled clinical trial	To investigate the effectiveness of a workstation ergonomic intervention for work-related posture and low back pain in video display terminal workers.	REBA: to assess posture, load or strength, repetitiveness and frequency of changing positions and drawing-based pain assessments (to indicate the extent of pain)	Ergonomic assessment, changes to design of workstation and physiotherapy assessment	The intervention improved work-related posture and was effective for reducing the prevalence of lumbar pain.

BPI = Brief Pain Inventory; CIS20-R = Checklist Individual Strength; VAS = visual analog scale; HWQ = Health and Work Questionnaire; MAF = Multidimensional Assessment of Fatigue Scale; NMQ = Nordic Musculoskeletal Questionnaire; OEA = Office Environment Assessment; REBA = Rapid Entire Body Assessment; RMDQ = Roland Morris Disability Questionnaire; RULA = Rapid Upper Limb Assessment.

**Chart 4:** Characteristics of studies in the category: use of auxiliary devices that could interfere with position and with execution of work tasks

Authors (year)	Sample (n)	Study design	Objective	Analytical instrument	Intervention	Outcome
Smith et al.^25^ (2015)	22 workers (men and women)	Quasi-experimental pilot study	Assess the impact of introduction of an alternative ergonomically-correct keyboard on perceptions: design, acceptability, usability, body discomfort, and typing productivity	BMI; Likert scale instrument to collect participants’ perceptions of design, acceptability and usability associated with the standard keyboard; self-report body discomfort: 8 cm scale for analysis of intensity in different body parts	Use of normal keyboard (currently used in workplace) or use of ergonomically-correct alternative keyboard	Workers stated that they liked the new keyboard and it was associated with reduction in body discomfort. No changes in typing performance were observed.
Joines et al.^26^ (2015)	95 workers (10 men and 85 women)	Randomized controlled clinical trial	To assess the ergonomic and calculated power consumption benefits of adjustable LED lighting in an office environment	Online questionnaire: to collect demographic information and reports of discomfort, level of eye fatigue, and perceptions of work content; assessment of each participant’s workspace: to record illumination level; RULA: to assess posture	Adjustable LED lighting	There were benefits for participants’ musculoskeletal comfort, posture, and visual comfort.
Hayes et al.^28^ (2016)	29 workers, the majority women (12 dental hygienists, 17 students)	Uncontrolled clinical trial	To investigate the effect of the use of loupes on neck pain and disability in dental hygienists	NPDS: to assess self-report pain and neck dysfunction scores; physical examination: to assess objective measures of musculoskeletal symptoms in the upper body	Use of loupes to magnify workers’ visual field	There was an increase in cervical movement amplitude and muscle resistance in the neck region. There was no significant difference between those using loupes and the control group in reduction of musculoskeletal pain.
Aghilinejad et al.^27^ (2016)	105 workers (all men)	Quasi-experimental study	To implement an interventional ergonomic program to minimize musculoskeletal discomfort in semiconductor industry assembly workers.	NMQ: to determine the prevalence of musculoskeletal symptoms; Corlett & Bishop body part discomfort scale: to assess discomfort	Using magnifying loupes to improve workers’ visual acuity.	Using the loupes reduced body discomfort among the workers, minimizing musculoskeletal disorders.

BMI = body mass index; NMQ = Nordic Musculoskeletal Questionnaire; NPDS = Neck Pain and Disability Scale; RULA = Rapid Upper Limb Assessment; LED = light emitting diode.

## DISCUSSION

Considering the studies included in this review, it was observed that several different ergonomic interventions yielded satisfactory results for workers, both with regard to posture and to musculoskeletal symptoms.^15-28^

Only one study was found that covered physical exercises with workers who work in a sitting position. However, its methodological quality was not high, although these practices are considered the gold standard intervention. This study found that after the after less than 2 months of the program of physical exercise and postural and ergonomic guidance, workers reported that pain and musculoskeletal discomfort had reduced, even among those without job satisfaction.^19^ Job satisfaction is a complex factor involving psychosocial aspects and workers’ mental health and wellbeing,^29^ and should be cared for and assessed in depth.

One study observed that daily occupational gymnastics in the workplace can improve gains in flexibility, reduce rates of physician-certified sick leave for low back pain and other pains^30^ and minimize reports and intensity of musculoskeletal pain.^31^ It could be inferred that inclusion of physical exercises with instruction from trained professionals works to encourage workers to adopt healthy measures in the workplace, in addition to encouraging them to do the exercises daily. Another relevant question is whether employers can encourage these practices with group or individual activities,^32^ contributing even more to improve physical condition and job satisfaction.

Four studies employed different types of guidance and instruments to facilitate adherence to the guidance,^16,17,20,21^ two of which had high methodological quality, using electronic biofeedback devices and reported positive results with reduction of musculoskeletal symptoms^16,17^ and improved posture among the workers.^16^

Use of instruments, questionnaires, protocols, and sensors enables working conditions and repetitiveness of tasks and postures to be assessed, helping to improve the working environment and working conditions.^33^ Along the same lines, a study that employed a biofeedback device with six individuals for a 5-hour period was able to demonstrate a contribution to maintenance of good posture while working on a computer.^34^

Only two of the studies about furniture configuration had high methodological quality, both controlled and randomized clinical trials,^15,18^ while all of the others scored below 7.^22-24^ These interventions contribute to a healthier, more flexible, more comfortable, more productive, and more satisfactory workplace.^15,18,22-24^ It was possible to identify reductions in low back pain among workers who used sitting workstations,^23^ after using sit-stand workstations,^18-24^ and after adjustments and modifications to office furniture,^15^ reporting improvements in postural aspects, reductions in musculoskeletal complaints, improved psychosocial conditions, and better disposition and interaction between workers in the work environment.^15,22-24^

All of the studies that employed auxiliary devices had methodological quality scores below 7. Use of these devices was effective for reduction of body discomfort, for improving posture, and for reducing musculoskeletal symptoms.^25,26^ One of the studies that employed interventions using magnifying loupes to improve workers’ vision identified significant reductions in discomfort involving the neck, shoulder, upper arm, elbows, lower arms, lower back, and whole body after the intervention and also improvements over time in cervical movement amplitude and in deep neck muscle resistance, although there were deteriorations in front head posture and cervical kinesthetic sensitivity, suggesting that the loupes have both positive and negative results for workers’ physical wellbeing.^28^

Workstations should be adaptable to each worker^35^ to enable greater efficiency and better satisfaction for the users who work with these systems,^36^ and ergonomic interventions can contribute to helping workers learn to adopt healthy postures when working^16^ and when concentrating on their work.^23^

The sitting position predominates in many types of jobs and remaining seated for prolonged periods can overload musculoskeletal structures and lead to development of symptoms such as pain and discomfort, since no posture is healthy if maintained for long periods and sitting should be a dynamic matter.^30^

Therefore, ergonomic interventions work to change workplace situations, contributing to improve physical conditions and helping to improve the tasks being undertaken, making them more appropriate for the workers performing them.^28^

## CONCLUSIONS

The studies demonstrate that, in general, the different ergonomic interventions were effective for reducing musculoskeletal symptoms in workers who work in a sitting position.

## References

[1] Abrahão JI (2000). Reestruturação produtiva e variabilidade do
trabalho: uma abordagem da ergonomia. Psicol Teor Pesqui.

[2] Meira-Mascarenhas CH, Ornellas-Prado F, Henrique-Fernandes M (2012). Dor musculoesquelética e qualidade de vida em agentes
comunitários de saúde. Rev Salud Publica (Bogota).

[3] Toscano JJO, Egypto EP (2001). A influência do sedentarismo na prevalência de
lombalgia. Rev Bras Med Esporte.

[4] Szeto GPY, Lam P (2007). Work-related musculoskeletal disorders in urban bus drivers of
Hong Kong. J Occup Rehabil.

[5] Marques NR, Hallal CZ, Gonçalves M (2010). Características biomecânicas, ergonômicas e
clínicas da postura sentada: uma revisão. Fisioter Pesqui.

[6] Kroemer KHE, Grandjean E (2005). Manual de ergonomia: adaptando o trabalho ao homem.

[7] Martins MRI, Foss MHDA, Santos Jr R, Zancheta M, Pires IC, Cunha AMR (2010). A eficácia da conduta do Grupo de Postura em pacientes com
lombalgia crônica. Rev Dor.

[8] Picoloto D, Silveira E (2008). Prevalência de sintomas osteomusculares e fatores
associados em trabalhadores de uma indústria metalúrgica de
Canoas - RS. Cienc Saude Colet.

[9] Associação Brasileira de Ergonomia (ABERGO) (2003). Norma ERG BR 1000: estabelecimento do Organismo Certificador do
Ergonomista Brasileiro (OCEB) [Internet].

[10] Martins CDO (2000). Efeitos da ginástica laboral em servidores da reitoria da
UFSC.

[11] Mendes KDS, Silveira RCCP, Galvão CM (2008). Revisão integrativa: método de pesquisa para a
incorporação de evidências na saúde e na
enfermagem. Texto Contexto Enferm.

[12] Souza MT, Silva MD, Carvalho R (2010). Revisão integrativa: o que é e como
fazer. Einstein (São Paulo).

[13] Santos CMC, Pimenta CAM, Nobre MRC (2007). A estratégia PICO para a construção da
pergunta de pesquisa e busca de evidências. Rev Latino-Am Enferm.

[14] The George Institute for Global Health Centre for evidence-based physiotherapy - PEDro: physiotherapy evidence
database [Internet].

[15] Pillastrini P, Mugnai R, Bertozzi L, Costi S, Curti S, Guccione A (2010). Effectiveness of an ergonomic intervention on work-related
posture and low back pain in video display terminal operators: a 3 years
cross-over trial. Appl Ergon.

[16] Bazazan A, Dianat I, Feizollahi N, Mombeini Z, Shirazi AM, Castellucci HI (2019). Effect of a posture correction-based intervention on
musculoskeletal symptoms and fatigue among control room
operators. Appl Ergon.

[17] Levanon Y, Gefen A, Lerman Y, Givon U, Ratzon NZ (2012). Multi dimensional system for evaluating preventive program for
upper extremity disorders among computer operators. Work.

[18] Ognibene GT, Torres W, von Eyben R, Horst KC (2016). Impact of a sit-stand workstation on chronic low back pain:
results of a randomized Trial. Int J Occup Environ Med.

[19] Martins LV, Baú LMS, Marziale MHP, Franco BAS (2011). Exercícios físicos e seus efeitos nas queixas
osteomusculares e na satisfação do trabalho. Rev Enferm.

[20] Ailneni RC, Syamala KR, Kim IS, Hwang J (2019). Influence of the wearable posture correction sensor on head and
neck posture: Sitting and standing workstations. Work.

[21] Yoo WG, Park SY (2015). Effects of posture-related auditory cueing (PAC) program on
muscles activities and kinematics of the neck and trunk during computer
work. Work.

[22] Robertson MM, Huang YH, Lee J (2017). Improvements in musculoskeletal health and computing behaviors:
effects of a macroergonomics office workplace and training
intervention. Appl Ergon.

[23] Dutta N, Walton T, Pereira MA (2015). Experience of switching from a traditional sitting workstation to
a sit-stand workstation in sedentary office workers. Work.

[24] Thorp AA, Kingwell BA, Owen N, Dunstan DW (2014). Breaking up workplace sitting time with intermittent standing
bouts improves fatigue and musculoskeletal discomfort in overweight/obese
office workers. Occup Environ Med.

[25] Smith ML, Pickens AW, Ahn S, Ory MG, DeJoy DM, Young K (2015). Typing performance and body discomfort among overweight and obese
office workers: a pilot study of keyboard modification. Appl Ergon.

[26] Joines S, James T, Liu S, Wang W, Dunn R, Cohen S (2015). Adjustable task lighting: Field study assesses the benefits in an
office environment. Work.

[27] Aghilinejad M, Azar NS, Ghasemi MS, Dehghan N, Mokamelkhah EK (2016). An ergonomic intervention to reduce musculoskeletal discomfort
among semiconductor assembly workers. Work.

[28] Hayes MJ, Osmotherly PG, Taylor JA, Smith DR, Ho A (2016). The effect of loupes on neck pain and disability among dental
hygienists. Work.

[29] Martinez MC, Paraguay AIBB, Latorre MRDO (2004). Relação entre satisfação com aspectos
psicossociais e saúde dos trabalhadores. Rev Saude Publica.

[30] Reis PF, Moro ARP, Contijo LA (2003). A importância da manutenção de bons
níveis de flexibilidade nos trabalhadores que executam suas
atividades laborais sentados. Rev Prod Online.

[31] Lima VA, Aquilas AL, Ferreira Jr M (2009). Efeitos de um programa de exercícios físicos no
local de trabalho sobre a percepção de dor
musculoesquelética em trabalhadores de
escritório. Rev Bras Med Trab.

[32] Carvalho TN, Lessa MR Sedentarismo no ambiente de trabalho: os prejuízos da postura
sentada por longos períodos [Internet].

[33] Serranheira F, Uva AS, Espírito-Santo J (2009). Estratégia de avaliação do risco de
lesões músculo-esqueléticas de membros superiores
ligadas ao trabalho aplicada na indústria de abate e desmancha de
carne em Portugal. Rev Bras Saude Ocup.

[34] Breen PP, Nisar A, Olaighin G (2009). Evaluation of a single accelerometer based biofeedback system for
real-time correction of neck posture in computer users. Annu Int Conf IEEE Eng Med Biol Soc.

[35] Thetkathuek A, Meepradit P (2018). Work-related musculoskeletal disorders among workers in an MDF
furniture factory in eastern Thailand. Int J Occup Saf Ergon.

[36] Cybis W, Betiol AH, Faut R (2015). Ergonomia e usabilidade.

